# Berbamine postconditioning protects the heart from ischemia/reperfusion injury through modulation of autophagy

**DOI:** 10.1038/cddis.2017.7

**Published:** 2017-02-02

**Authors:** Yanjun Zheng, Shanshan Gu, Xuxia Li, Jiliang Tan, Shenyan Liu, Yukun Jiang, Caimei Zhang, Ling Gao, Huang-Tian Yang

**Affiliations:** 1 Key Laboratory of Stem Cell Biology and Laboratory of Molecular Cardiology, Institute of Health Sciences, Shanghai Jiao Tong University School of Medicine & Shanghai Institutes for Biological Sciences, Chinese Academy of Sciences, Shanghai, China; 2 Heart Center, Shanghai Jiaotong University Affiliated Sixth People's Hospital, Shanghai, China

## Abstract

Pretreatment of berbamine protects the heart from ischemia/reperfusion (I/R) injury. However it is unknown whether it has cardioprotection when given at the onset of reperfusion (postconditioning (PoC)), a protocol with more clinical impact. Autophagy is upregulated in I/R myocardium and exacerbates cardiomyocyte death during reperfusion. However, it is unknown whether the autophagy during reperfusion is regulated by berbamine. Here we investigated whether berbamine PoC (BMPoC) protects the heart through regulation of autophagy by analyzing the effects of BMPoC on infarct size and/or cell death, functional recovery and autophagy in perfused rat hearts and isolated cardiomyocytes subjected to I/R. Berbamine from 10 to 100 nM given during the first 5 min of reperfusion concentration-dependently improved post-ischemic myocardial function and attenuated cell death. Similar protections were observed in cardiomyocytes subjected to simulated I/R. Meanwhile, BMPoC prevented I/R-induced impairment of autophagosome processing in cardiomyocytes, characterized by increased LC3-II level and GFP-LC3 puncta, and decreased p62 degradation. Besides, lysosomal inhibitor chloroquine did not induce additional increase of LC3-II and P62 abundance after I/R but it reversed the effects of BMPoC in those parameters in cardiomyocytes, suggesting that I/R-impaired autophagic flux is restored by BMPoC. Moreover, I/R injury was accompanied by enhanced expression of Beclin 1, which was significantly inhibited by BMPoC. *In vitro* and *in vivo* adenovirus-mediated knockdown of Beclin 1 in myocardium and cardiomyocytes restored I/R-impaired autophagosome processing, associated with an improvement of post-ischemic recovery of myocardial contractile function and a reduction of cell death, but it did not have additive effects to BMPoC. Conversely, overexpression of Beclin 1 abolished the cardioprotection of BMPoC as did by overexpression of an essential autophagy gene *Atg5*. Furthermore, BMPoC-mediated cardioprotection was abolished by a specific Akt1/2 inhibitor A6730. Our results demonstrate that BMPoC confers cardioprotection by modulating autophagy during reperfusion through the activation of PI3K/Akt signaling pathway.

Myocardial dysfunction resulting from ischemia/reperfusion (I/R) is a common clinical scenario in patients suffering from ischemic heart disease. Therefore, during last four decades, the novel strategies to attenuate lethal myocardial I/R injury have been intensively investigated.^1, 2, 3, 4, 5^ Ischemic preconditioning (a short sequence of I/R before an index prolonged ischemia) is a protective strategy by showing the attenuation of lethal reperfusion injury.^6, 7^ Afterwards, Zhao *et al.*^8^ demonstrated that repetitive short period of I/R applied during early reperfusion (postconditioning (PoC)) significantly reduce myocardial injury. This approach is operative in all species including humans, although the mechanisms need to be further explored.^9, 10, 11, 12^ Meanwhile several pharmacological interference during late infarction, immediately prior to reperfusion or at the onset of reperfusion have been tested.^13, 14, 15^ This approach is more clinically feasible than mechanical PoC, while so far limit agents are clinically available for the patient with ischemic heart disease.^16, 17^

Traditional Chinese medicine (TCM) has been practiced for thousands of years, providing a vast source of pharmaceutical materials. Berbamine is a natural small-molecule compound, derived from the roots, barks and stems of Barberry which has been used as a medicinal plant in TCM. Berbamine has anti-tumor, immunomodulatory and cardiovascular effects, and has been used to treat patients with a low level of white blood cells caused by chemotherapy and/or radiotherapy.^18, 19, 20^ We recently found that it has a positive inotropic effect when used within a lower concentration range on the rat heart.^21^ Moreover, we found that its preconditioning confers cardioprotective effects against I/R injury through the maintenance of cytosolic Ca^2+^ homeostasis and the inhibition of calpain activation.^22^ However, it is unknown whether it has cardioprotection when given at the onset of reperfusion (berbamine PoC (BMPoC)), an approach with more clinical impact.

Autophagy or more precisely macroautophagy is a tightly regulated intracellular catabolic process that serves as the cellular quality control mechanisms for the disposal of damaged and dysfunctional organelles and protein aggregates, thus it is widely implicated in pathophysiological processes, including cardiovascular diseases.^23, 24^ Autophagy is up-regulated in I/R myocardium and it is detrimental and exacerbates cardiomyocyte death during reperfusion,^25^ although some reports showed that autophagy is cardioprotective.^26, 27^ It has been shown that Beclin 1 is a key autophagic protein regulating both autophagosome formation and processing and the up-regulation of Beclin 1 is responsible for autophagy activation and cell death during reperfusion.^28, 29^ *Beclin* 1 heterozygous knockout (*beclin* 1^+/−^) mice significantly decreases the autophgosome formation and reduces the infarct size.^30^ However, it is unknown whether and how berbamine regulates the autophagy during reperfusion.

To address these issues, this study was designed to (i) characterize the concentration-dependent effects of BMPoC on the myocardial I/R injury; (ii) clarify the effects of berbamine on the autophagy during I/R; (iii) determine the involvement of Beclin 1 in the BMPoC-afforded cardioprotective effects; and (iv) explore the underlying signaling pathway that contributes to the BMPoC-induced cardioprotection. Our results provide new insight into the mechanism of BMPoC-induced cardioprotection and suggest a potential application value of berbamine in the protection of hearts against I/R injury.

## Results

### BMPoC improves post-ischemic recovery of myocardial performance and cell survival

**Figure 1:**
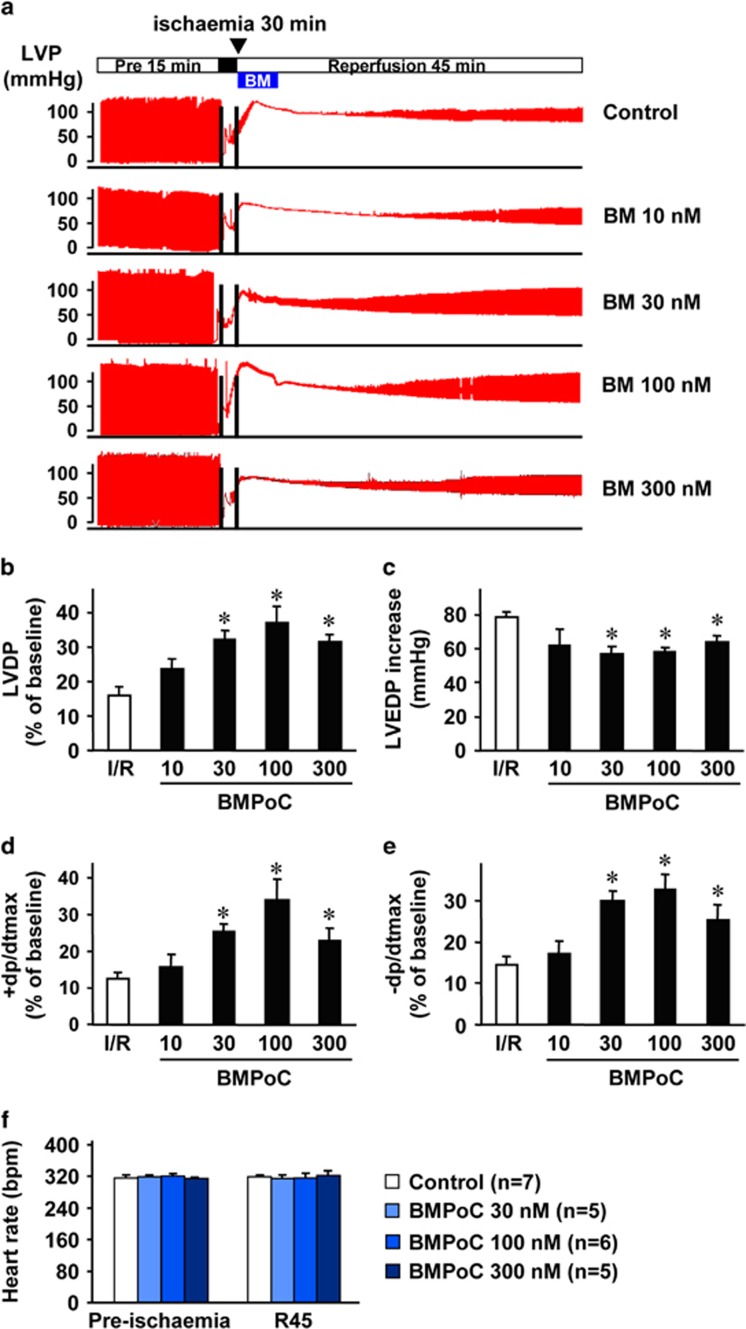
Effect of BMPoC on the LV functional recovery. Hearts were exposed to berbamine at various concentrations (control *n*=7; 10 nmol *n*=5; 30 nmol *n*=5; 100 nmol *n*=6; 300 nmol *n*=5) for 5 min at the onset of reperfusion. (**a**) Representative traces of LV pressure (LVP); (**b**–**e**) The post-ischemic recovery of LVDP (**b**),LVEDP (**c**), maximum rate of pressure development over time (+dp/dtmax, **d**); maximum rate of pressure decay over time (−dp/dtmax, **e**); (**f**) Heart rate at 5 min prior to ischemia (Pre-ischemia) and 45 min of reperfusion (R45) with and without BMPoC. ▾, start time point for the addition of berbamine; control, without BMPoC. Data were presented as means±S.E.M. *P<0.05 *versus* I/R control

To determine the cardioprotective effects of BMPoC against I/R injury, we perfused isolated rat hearts with berbamine at concentrations from 10 to 300 nM during the first 5 min of reperfusion. The contractile function of left ventricular (LV), including LV developed pressure (LVDP), LV end-diastolic pressure (LVEDP), maximal rates of pressure development over time (+dP/dtmax) and pressure decay over time (−dP/dtmax), was severely suppressed during 45 min reperfusion following 30 min ischemia (Figures 1a–e). BMPoC did not affect the heart rate at reperfusion within the concentration range we examined (Figure 1f), while it concentration-dependently improved the I/R-suppressed post-ischemic myocardial performance from 10 to 100 nM and did not have a further improvement at 300 nM (Figures 1a–e).

**Figure 2:**
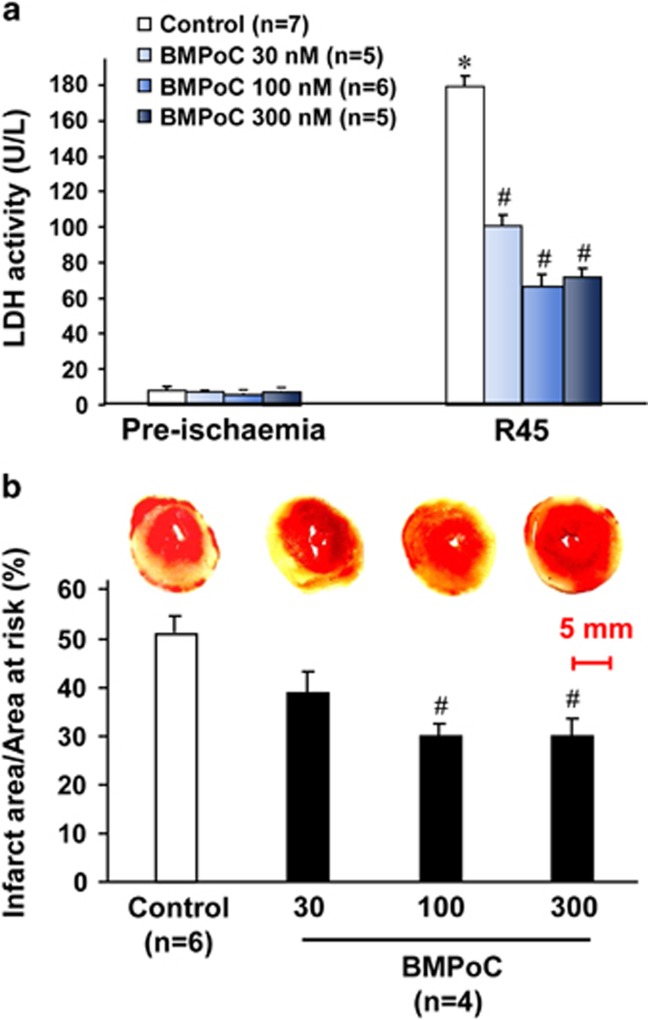
Effect of BMPoC on the LDH activity in the coronary effluent and LV infarct size in isolated rat hearts subjected to I/R.(**a**) LDH activity in coronary effluent, (**b**) Representative images and analysis of the infarct size in isolated I/R (30 min/2 h) hearts with or without 100 nM berbamine. R45, 45 min of reperfusion. Data were presented as means±S.E.M. *P<0.05 *versus* pre-ischemic control; ^#^*P*<0.05 *versus* I/R control

Next, we investigated whether berbamine affects cell survival by detecting lactate dehydrogenase (LDH) release, an indicator of myocardial injury,^31^ and myocardial infarct size in isolated hearts suffering 30 min of ischemia followed by 120 min of reperfusion. The LDH release was hardly detected in the coronary effluent during pre-ischemia in both control and berbamine groups and it was markedly induced at the end of reperfusion, while the increase was significantly suppressed by BMPoC from 30 to 300 nM (Figure 2a). Consistently, the I/R-induced infarction after 2 h of reperfusion was significantly attenuated by BMPoC from 30 to 300 nM (Figure 2b).

### BMPoC improves cell survival, contraction and mitochondrial membrane potential in simulated I/R cardiomyocytes

**Figure 3:**
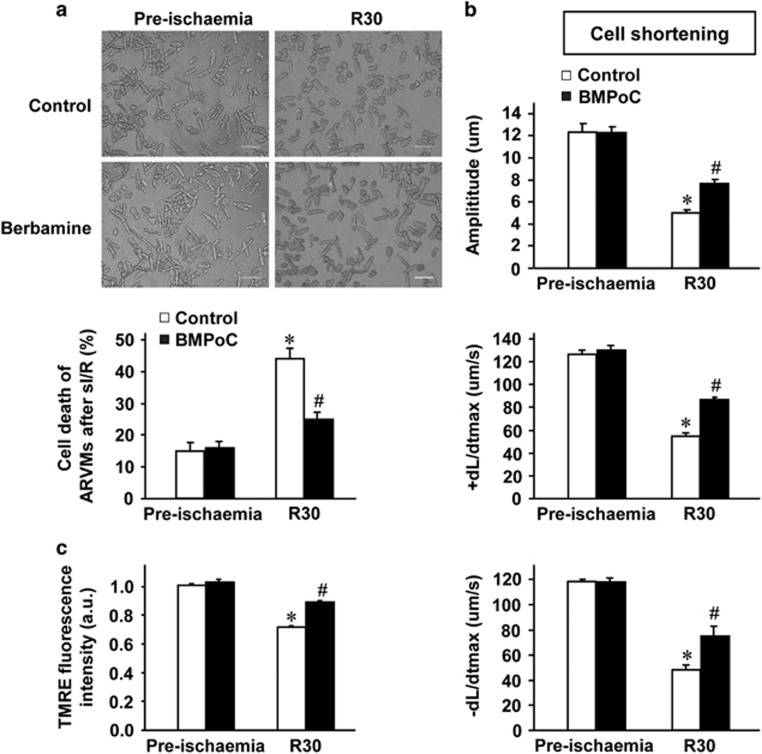
Effects of BMPoC on the cell death, cell shortening and dissipation of mitochondrial membrane potential (ΔΨ_m_) induced by simulated I/R in isolated rat cardiomyocytes. (**a**) Representative images of cardiomyocytes morphology (upper panel) and quantitative percentages (lower panel) of cell death with and without simulated I/R in the presence and absence of berbamine (30 nM); (**b**) Analysis of the amplitude and maximum speed of cell shortening/re-lengthening (±dL/dtmax); (**c**) Summarized data of TMRE-indicated ΔΨ_m_ with various concentrations of BMPoC. Scale bar=100 *μ*m. The statistical analysis was conducted on the averaged value for each rat heart and *n*=5; data were presented as means±S.E.M.; *P<0.05 *versus* the pre-ischemic control; ^#^*P*<0.05 *versus* the simulated I/R control

To determine whether berbamine confers cardioprotection through its direct action on the cardiomyocytes, we subjected the isolated cardiomyocytes to simulated I/R and applied berbamine to cells during the first 5 min of reperfusion. In line with the effects of berbamine on the myocardial I/R injury, simulated I/R-induced cell death was significantly attenuated by BMPoC (Figure 3a). Moreover, simulated I/R-depressed the amplitude and maximum speed of cell shortening/re-lengthening (±dl/dtmax) were significantly attenuated by 30 nM of BMPoC (Figure 3b). In addition, simulated I/R-decreased the intensity of tetramethylrhodamine ethyl ester (TMRE) fluorescence, which reflects the depolarization of ΔΨm (mitochondrial membrane potential, indicated with TMRE, a critical event in the process of cell death^32^), was preserved by BMPoC (Figure 3c).

### BMPoC recovers I/R-impaired autophagic flux

**Figure 4:**
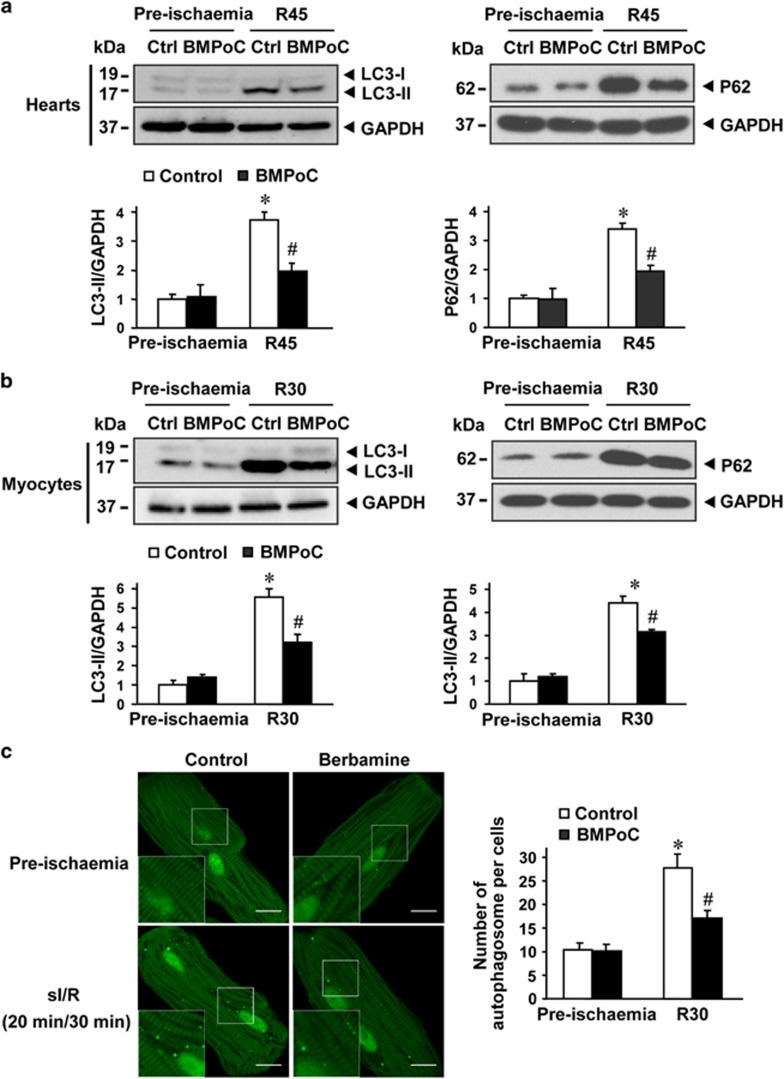
Effect of berbamine (BM) on autophagy in isolated rat hearts and adult rat ventricular cardiomyocytes subjected to I/R. (**a**and**b**) Representative immunoblots (left panel) and quantitative analysis (right panel) of light chain-3 (LC3) (left panel) and P62 (right panel) in myocardial extracts (**a**) and cardiomyocyte lysis (**b**) subjected to I/R in the presence or absence of BMPoC; (**c**) representative (left panel) and analysis (right panel) of immunofluorescence images of cardiomyocytes expressing green fluorescent protein-light chain-3 (LC3) and LC3 puncta (indicator of autophagosomes) in cardiomyocytes after 36 h infection of Ad-GFP-LC3 (100 multiplicities of infection) with and without simulated I/R. Ctrl, control. Scale bar=10 *μ*m. The statistical analysis was conducted on the averaged value for each rat heart and *n*=5; data were presented as means±S.E.M. *P<0.05 *versus* pre-ischemic control; ^#^*P*<0.05 *versus* the corresponding I/R control

Next, whether and how autophagy plays a role in BMPoC-afforded cardioprotection were examined. Both I/R hearts (Figure 4a, left panel) and cardiomyocytes (Figure 4b, left panel) significantly increased the LC3-II abundance, while it was reversed by BMPoC. Then the cardiomyocytes infected with adenovirus encoding GFP-LC3 showed a punctate staining pattern of GFP-LC3 in simulated I/R, while the I/R-increased dots of GFP-LC3 were significantly reduced by BMPoC (Figure 4c). Meanwhile, the expression of P62, a specific autophagic substrate protein and the hallmark representing autophagic flux,^28^ was markedly increased in both I/R hearts (Figure 4a, right panel) and cardiomyocytes (Figure 4b, right panel), while these alternations were attenuated by BMPoC. To further confirm the effects of I/R and BMPoC on the autophagic flux, we used chloroquine (CQ) to prevent autophagosome-lysosome fusion.^28^ CQ did not induce additional increase LC3-II abundance and P62 accumulation in adult cardiomyocytes subjected to simulated I/R, implicating a block of autophagic flux, but it reversed BMPoC-decreased LC3-II abundance and P62 accumulation (Supplementary Figure 1a). These results suggest that I/R-induced blockade of autophagic flux is recovered by BMPoC.

### BMPoC modulates I/R-induced autophagy and ΔΨm decrease via regulating Beclin 1

**Figure 5:**
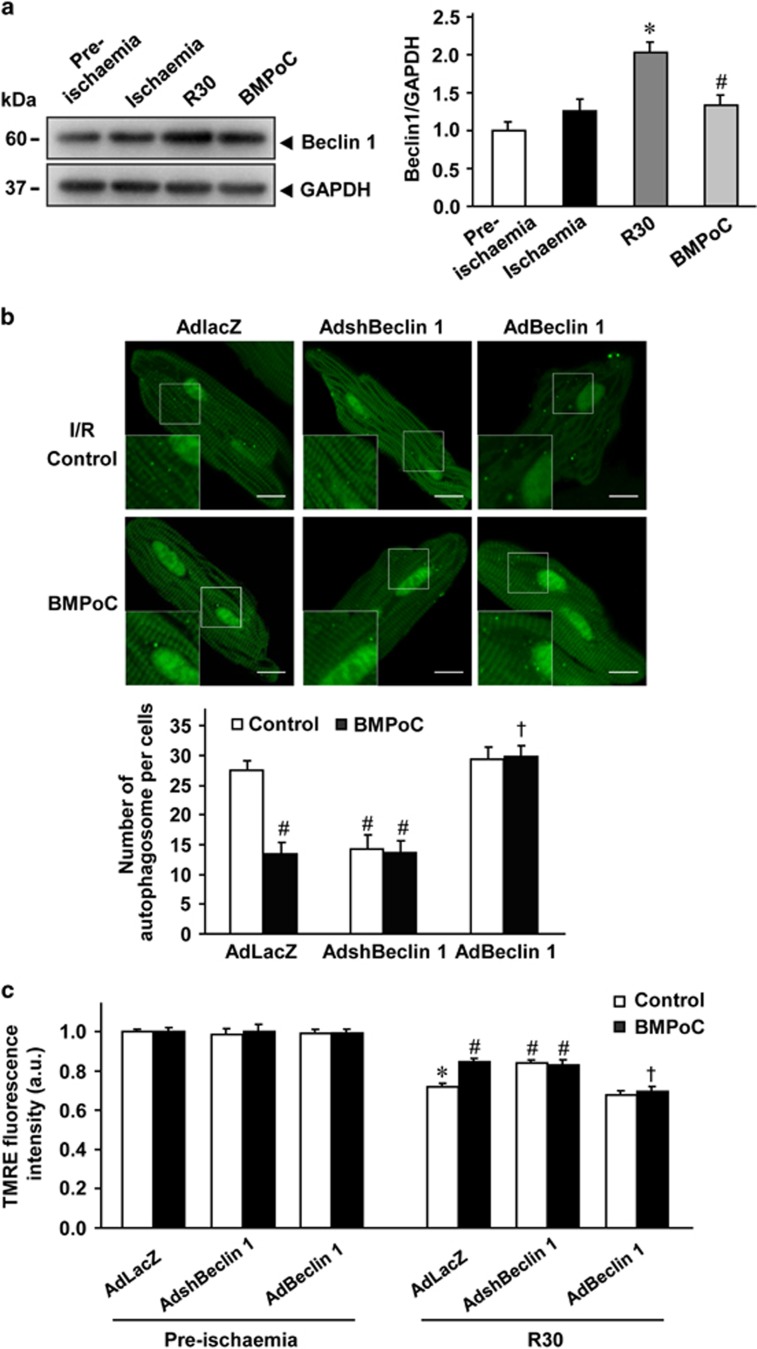
Effect of Beclin 1 on the autophagy and dissipation of ΔΨ_m_ induced by simulated I/R with or without berbamine. (**a**) Beclin 1 expressions during simulated I/R with or without BMPoC in cardiomyocytes. *n*=5. (**b**) Representative images (upper panel) and analysis (lower panel) of fluorescent GFP-LC3 puncta in simulated I/R cardiomyocytes at 30 min of reperfusion after Ad-GFP-LC3 infection. *n*=30–40 cells from 3 to 4 independent hearts. Scale bar=10 *μ*m. *versus* the AdLacZ-infected I/R hearts; (**c**) Summarized data of TMRE-indicated ΔΨ_m._ The statistical analysis was conducted on the averaged value for each rat heart and *n*=5; data were presented as means±S.E.M.. *P<0.05 *versus* the corresponding pre-ischemic groups; ^#^*P*<0.05 *versus* the corresponding control simulated I/R (R30) cardiomyocytes; ^†^*P*<0.05 *versus* the AdLacZ-infected cardiomyocytes with BMPoC

The autophagy-associated tumor suppressor gene, *Beclin 1*, has been reported to play an important role in the autophagic cell death due to I/R.^33^ To test whether the expression level of Beclin 1 is modulated by berbamine, we assessed mRNA and protein levels of Beclin 1 in cardiomyocytes subjected to I/R. The Beclin 1 expression level in protein (Figure 5a) but not in mRNA (Supplementary Figure 2a) was significantly up-regulated in simulated I/R cardiomyocytes compared with the pre-ischemic ones, while the increase was significantly reduced by BMPoC (Figure 5a). The similar phenomenon was observed in I/R rat left ventricles (Supplementary Figures 2b and c), suggesting a possible involvement of Beclin 1 in I/R injury and BMPoC-afforded cardioprotection.

To clarify the role of Beclin 1 in the cardioprotective effect of BMPoC, we then measured the autophagic activity and ΔΨ_m_ in vehicle AdLacZ-, AdshBeclin 1- and AdBeclin 1-infected cardiomyocytes under simulated I/R with or without BMPoC. Beclin 1 protein levels increased ~1.76-fold in AdBeclin 1-infected cells and was ~2-fold down in AdshBeclin 1-infected cells related to AdLacZ control cells after 36 h of infection (Supplementary Figure 3a). Knockdown of Beclin 1 with AdshBeclin 1 infection resulted in the restoration of autophagosome processing as indicated by reduced GFP-LC3 puncta, LC3-II and P62 accumulation without an additive effect to the protective effect of BMPoC (Figure 5b and Supplementary Figure 1b), suggesting the modulation of BMPoC on I/R-induced autophagy via inhibiting Beclin 1. This was confirmed by Beclin 1 overexpression with AdBeclin 1 infection, which did not further enhance the I/R-increased GFP-LC3 puncta, LC3-II abundance and P62 expression but abolished the improvement of BMPoC on these parameters (Figure 5b and Supplementary Figure 1b). Meanwhile, the autophagosome number remained unchanged or comparable among vehicle AdLacZ-, AdshBeclin 1- and AdBeclin 1-infected cardiomyocytes (Supplementary Figure 4). Consistently, TMRE-indicated ΔΨ_m_ were similar among the AdLacZ-, AdshBeclin 1- and AdBeclin 1-infected cardiomyocytes during pre-ischemic phase with or without BMPoC, while they were reversed by AdshBeclin 1 as did by BMPoC but without the additive effect (Figure 5c). In contrast, overexpressed exogenous Beclin 1 in the cardiomyocytes abolished the protective effects of BMPoC against simulated I/R-impaired autophagosome processing and reduced ΔΨ_m_, although AdBeclin 1 did not alter simulated I/R-induced autophagic activity or reduced ΔΨ_m_ (Figures 5b and c). These data indicate that Beclin 1 contributes to the protection of BMPoC against I/R-induced autophagy dysfunction and ΔΨ_m_ decrease.

### BMPoC-improved post-ischemic recovery of contractile function and infarct size via suppressing Beclin 1 expression

**Figure 6:**
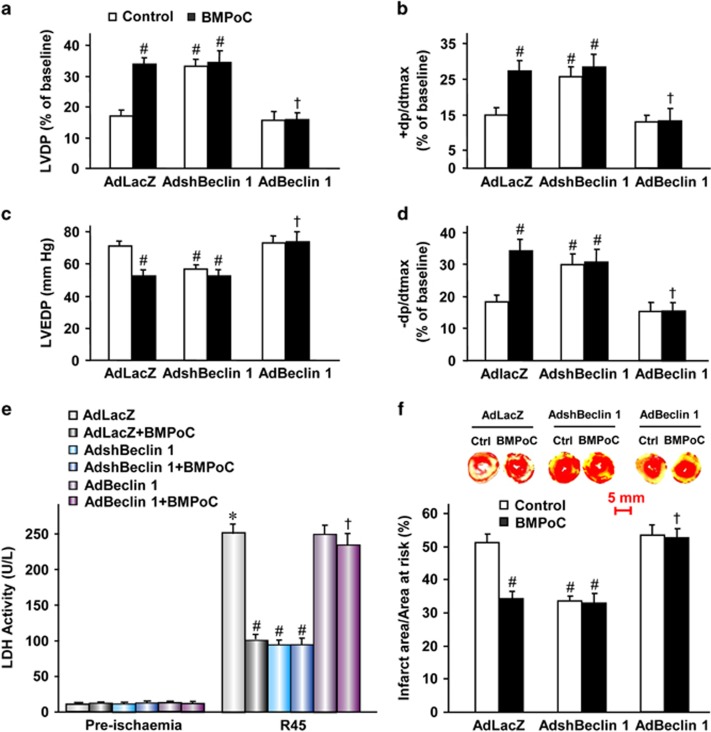
Effects of Beclin 1 on LV performance and cell death in isolated rat hearts subjected to I/R with or without BMPoC. (**a**–**d**)Effects of Beclin 1-knockdown and overexpression on the post-ischemic recovery of LVDP (**a**), +dp/dtmax (**b**), LVEDP (**c**) and −dp/dtmax (**d**) with or without BMPoC; (**e**) Effects of Beclin 1-knockdown and overexpression on the LDH activity in the coronary effluent with or without BMPoC; (**f**) Representative images (upper panel) and analysis (lower panel) of infarct size in perfused I/R (30 min/2 h) hearts. R45, 45 min of reperfusion. *n*=5 hearts each; data were presented as means ±S.E.M. *P<0.05 *versus* the pre-ischemic AdLacZ group; ^#^*P*<0.05 *versus* the AdLacZ-infected I/R hearts; ^†^*P*<0.05 *versus* the AdLacZ-infected hearts with BMPoC

We then investigated whether Beclin 1 contribute to BMPoC-afforded cardioprotection by manipulating Beclin 1 level in the hearts with *in vivo* adenoviral gene delivery. The expression level of Beclin 1 protein was a ~2-fold down in AdshBeclin 1-infected hearts and a ~1.9-fold increase in AdBeclin 1-infected hearts related to the AdLacZ hearts at day 3 after the injection of the adenovirus (Supplementary Figure 3b). No significant differences were observed in LVDP, LVEDP, ±dP/dt and HR among Langendorff-perfused rat hearts infected with AdLacZ control, AdshBeclin 1 and AdBeclin 1 during the pre-ischemic phase (Supplementary Figures 5a–d), while the I/R-suppressed post-ischemic recovery of contractile function in AdLacZ hearts was significantly improved by BMPoC (Figures 6a–d), although the HR was comparable among each groups at the reperfusion 45 min (Supplementary Figure 5e). The cardioprotection of BMPoC was mimicked by Beclin 1-knockdown. The contractile function of LVDP, LVEDP and ±dP/dt was comparable between the control and AdshBeclin 1 groups during pre-ischemic phase (Supplementary Figures 5a–d), while these parameters at post-ischemic phase were significantly improved in AdshBeclin 1 hearts compared with the AdLacZ hearts (Figures 6a–d), with a concurrent reduction of LDH activity in coronary perfusate at 45 min of reperfusion (Figure 6e) and myocardial infarct size after 2 h of reperfusion (Figure 6f). Moreover, Beclin 1-knockdown did not have an additive effect to BMPoC (Figures 6a–f). In contrast, Beclin 1 overexpression did not further enhanced the I/R-induced contractile dysfunction or infarct area, but it abolished the protective effect of BMPoC on these parameters after I/R (Figures 6a–d), although the HR remained comparable among the groups after I/R (Supplementary Figure 5e). Consistently, BMPoC-decreased LDH activity (Figure 6e) and infarct size (Figure 6f) due to I/R were significantly reversed by Beclin 1 overexpression, while AdBeclin 1 did not enhance the I/R-induced cell death. Having established that BMPoC modulates autophagy and confers cardioprotective effects, we next examined whether this protection is mainly dependent on the regulation of autophagy by overexpression of an essential autophagy gene *Atg5* through *in vivo* adenoviral gene delivery. Three days after the injection of the adenovirus-mediated Atg5 overexpression, the hearts were subjected to I/R with or without BMPoC. Similar with the beclin 1 overexpression, Atg5 overexpression abolished BMPoC-improved post-ischemic contractile function and reduced LDH activity in coronary perfusate at 45 min of reperfusion and infarct size at 2 h of reperfusion (Supplementary Figure 6), indicating that regulation of autophagy is responsible for cardioprotective effects of BMPoC. These results suggest that cardioprotective effects afforded by BMPoC are mediated through the inhibition of Beclin 1-induced autophagy during reperfusion, while the alternation of Beclin 1 levels within a certain extent seems not affect the basic myocardial function.

### PI3K/Akt signaling pathway is involved in the cardioprotective effects of BMPoC

**Figure 7:**
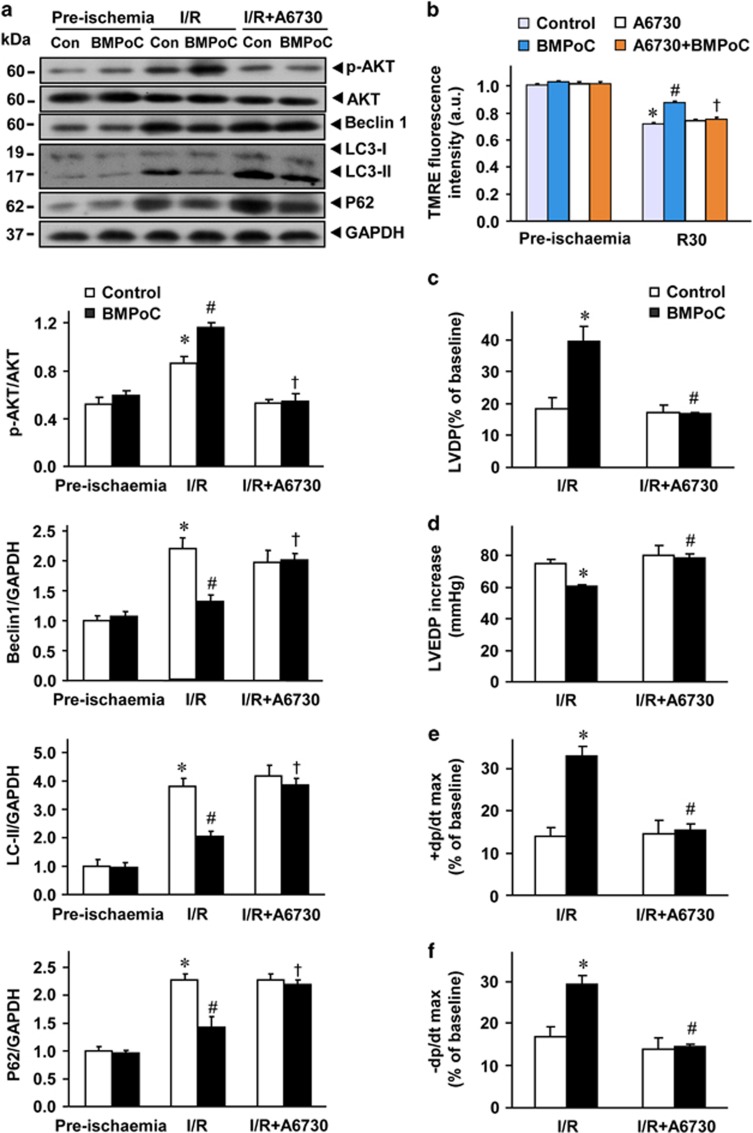
Activation of Akt contributes to BMPoC-restored I/R-altered autophagy and dissipation of mitochondrial membrane potential (ΔΨ_m_) as well as improved post-ischemic recovery of contractile function. (**a**) Representative immunoblots (upper panel) and averaged immunoblot data (lower panels) for the total and phosphorylation levels of Akt, Beclin 1, P62 and LC3-I, LC3-II. *n*=5; (**b**) Summarized data of TMRE-indicated ΔΨ_m_ of BMPoC with or without a specific Akt inhibitor A6730 (2.5 *μ*M); (**c**–**f**) effects of A6730 on the post-ischemic recovery of LVDP (**c**), +dp/dtmax (**d**), LVEDP (**e**), −dp/dtmax (**f**). *n*=4–6 hearts each; data were presented as means ±S.E.M. *P<0.05 *versus* the pre-ischemia control; ^#^*P*<0.05 *versus* the control I/R hearts; ^†^*P*<0.05 *versus* the I/R hearts with BMPoC

**Figure 8:**
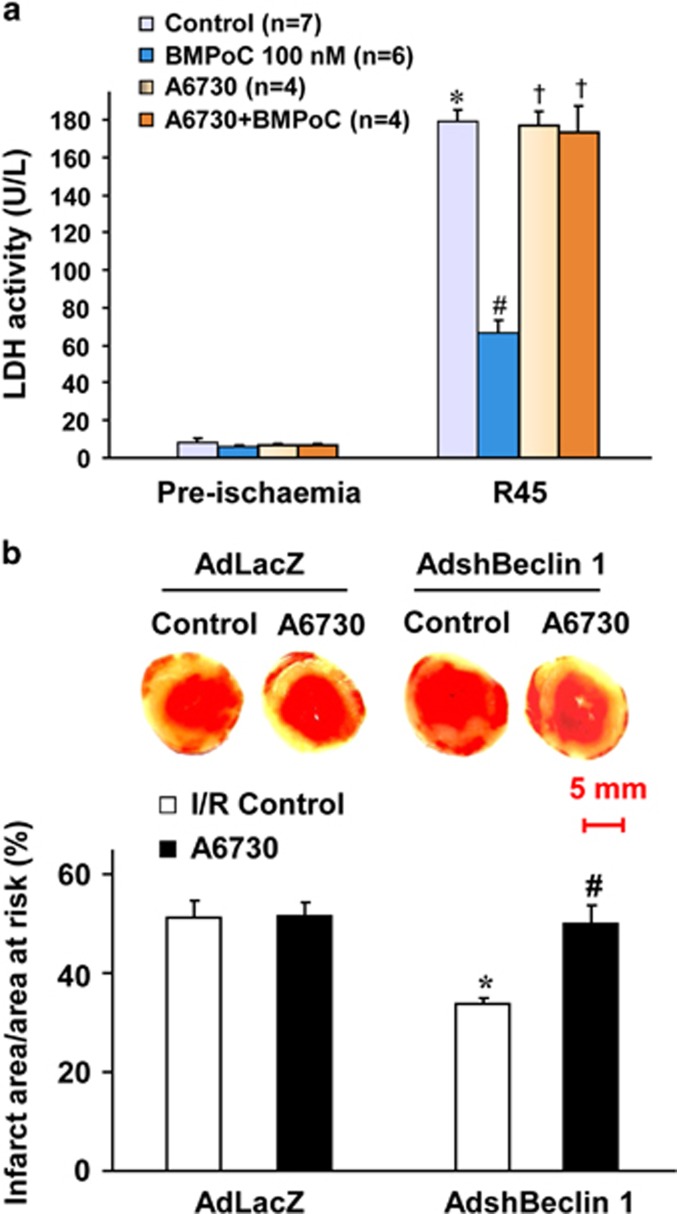
Effect of beclin 1 and Akt inhibitor A6730 on the LDH activity and LV infarct size. (**a**) LDH activity in coronary effluent collected at 45 min of reperfusion. Data were presented as means±S.E.M. *P<0.05 *versus* pre-ischemic control; ^#^*P*<0.05 *versus* I/R control; ^†^*P*<0.05 *versus* the I/R hearts with BMPoC at 100 nM. (**b**) Representative images (upper panel) and analysis (lower panel) of infarct size in isolated I/R (30 min/2 h) hearts. *n*=4–6 hearts each. Data were presented as means±S.E.M.; *P<0.05 *versus* the AdLacZ-infected I/R hearts; ^#^*P*<0.05 *versus* the AdshBeclin 1-infected I/R hearts

Next, we examined the signaling pathways that may mediate the inhibition of autophagy dysfunction-mediated cell death and pro-cell survival effects of berbamine. Western blotting analysis showed that I/R did not alter the expression level of total Akt, but the phosphorylation of Akt (Ser473) was increased in I/R myocardium compared with that of non-ischemia hearts, and it was further enhanced by BMPoC (Figure 7a). To further determine if the PI3K/Akt signaling pathway is involved in the BMPoC-mediated inhibition of Beclin 1 expression and subsequently contribute to the improvement of autophagosome processing as well as post-ischemic cardiac performance, a specific Akt inhibitor A6730^34^ was applied. A6730 at 2.5 *μ*M blocked BMPoC-increased Akt phosphorylation during reperfusion but not affected the total expression level of Akt (Figure 7a). It also abolished BMPoC-induced down-regulation of Beclin 1 and LC3-II expression as well as BMPoC-attenuated P62 accumulation during I/R (Figure 7a). BMPoC-protected ΔΨm were canceled by A6730 too in simulated I/R cells (Figure 7b). Moreover, A6730 did not affect the post-ischemic recovery of LVDP, LVEDP and ±dP/dt in the control group but it abolished BMPoC-improved myocardial contractile function (Figures 7c–f) and BMPoC-reduced LDH activity in coronary perfusate at 45 min of reperfusion (Figure 8a), although A6730 did not affect the heart rate at reperfusion with or without BMPoC (Supplementary Figure 7). In addition, A6730 abolished the cardioprotective effect of Beclin 1-knockdown in the infarct size (Figure 8b). These data support that BMPoC confers cardioprotective effects by suppressing Beclin 1-dependent autophagy dysfunction through the activation of PI3K/Akt signaling pathway.

## Discussion and Conclusion

In the present study, we demonstrated that (i) BMPoC concentration-dependently improves post-ischemic myocardial function; (ii) this protection is at least partially related to the improvement of cell survival and mitochondrial membrane potential by BMPoC in the I/R cardiomyocytes; (iii) I/R-enhanced Beclin 1 expression is significantly suppressed by BMPoC, which subsequently contributes to the BMPoC-restored autophagosome processing, improved myocardial function and reduced infarction during reperfusion; and (iv) the cardioprotective effects of BMPoC are mediated by the activation of PI3K/Akt signaling pathway. These results extend previous findings indicating the cardioprotection of BMPoC against I/R injury and reveal the new mechanisms of berbamine in the cardioprotection.

Reducing infarct size by pharmaceutical interventions as an adjunct to classical reperfusion interventions would be an attractive therapeutic principle.^35^ Our results showed that BMPoC from 10 to 100 nM improves post-ischemic myocardial function in a concentration-dependent manner and attenuates myocyte death (Figures 1 and 2). The protection is related to the direct action of BMPoC on the cardiomyocytes as BMPoC has similar improvement on the cell contraction in the simulated I/R cardiomyocytes (Figure 3). These data are consistent with the observation of cardioprotective effects of berbamine preconditioning we^22^ and others^36, 37^ reported. Notably, the concentrations of berbamine-afforded cardioprotection are within the range of inducing positive inotropic effects in the rat hearts but are much lower than those causing negative effects in the isolated guinea pig isolated hearts.^21, 38^ Therefore, berbamine may be benefit to patients with myocardial contractile dysfunction either with or without I/R injury.

In addition to the direct improvement of BMPoC in post-ischemic cell contraction, BMPoC significantly limits the infarct size by reducing the I/R-induced cell death (Figures 2 and 3a). This effect should also contribute to the BMPoC-afforded protection on the post-ischemic myocardial function. Interestingly, the protection of berbamine is mediated by modulating autophagy and decreasing autophagy-mediated cell death in the setting of I/R injury. This is supported by following evidence: (i) BMPoC attenuates I/R-induced impairment of autophagosome processing both in myocardium and cardiomyocytes (Figures 4 and 5 and Supplementary Figure 1a); (ii) BMPoC inhibits I/R-enhanced expression of Beclin 1, an autophagy-related gene, in I/R myocardium (Figure 5a) and cardiomyocytes (Supplementary Figure 2b and Figure 7a); and (iii) knockdown of Beclin 1 attenuates I/R-induced autophagy dysfunction and recovers autophagic flux but without an additive effect to the protection of BMPoC, while Beclin 1 as well as an essential autophagy gene *Atg5* overexpression abolish the improvement of BMPoC on these parameters (Supplementary Figure 1b). Meanwhile the significant induction of autophagy and impaired autophagy flux in I/R hearts and in simulated I/R ventricular cardiomyocytes (Figures 4a and b) are consistent with the previous reports,^30, 39, 40^ although how the level of beclin1 is affected by various interventions *in vivo* needs to be investigated.

The increased autophagy during the ischemic phase is demonstrated to be protective, while the functional role of autophagy during the reperfusion has not yet been fully clarified.^28, 30, 41, 42^ It has been shown that the induction of autophagy during the reperfusion is detrimental.^30, 39^ This is supported by our data showing that the impaired autophagosome processing in I/R myocardium is associated with the post-ischemic contractile dysfunction and cell death, while the modulation of autophagy by BMPoC is cardioprotective (Figure 6). However, other reports showed that the stimulation of autophagy during reperfusion is cardioprotective.^26, 27^ The contradictory results may be interpreted by the different cell types, animal species and I/R models. For example, the autophagy constitutes a protective mechanism against simulated I/R injury in HL-1 cells^26^ or in remote limb ischemic PoC of mice.^27^ The different cell types and ischemic PoC models may have differential downstream pathways triggered by autophagy which weave a complex interplay of responses that may be either beneficial or detrimental. Our results reveal that the pharmacological PoC confers cardioprotective effects via the modulation of autophagy during myocardial reperfusion which has not yet been well recognized.

Beclin 1 is one particularly interesting autophagy-related gene which forms a protein complex with a phosphatidyl inositol-3-kinase within the autophagosome, and Beclin 1-deficient mice show a pronounced loss of autophagic vacuole formation.^43^ It has also been shown that Beclin 1-dependent autophagy is involved in various pathophysiological processes.^30, 44^ Up-regulation of Beclin 1 seems to play an important role in the mediation of autophagy during the reperfusion phase.^30^ Supportly, our data showed that a single cycle of I/R produces a marked increase in the expression of Beclin 1 in both cardiomyocytes (Figure 5a) and hearts (Supplementary Figures 2b and Figure 7a). I/R-upregulated Beclin 1 expression is significantly reduced by BMPoC in rat cardiomyocytes and left ventricles (Figure 5a and Supplementary Figure 2b), while Beclin 1-knockdown either in isolated cardiomyocytes or in the gene delivered whole hearts improves post-ischemic recovery of contractile function and limited infarct size (Figure 6). This is consistent with the previous observation that *beclin 1* heterozygous knockout mice attenuate the autophagy and myocardial injury.^30, 45^ Moreover, Beclin 1 overexpression in the isolated cardiomyocytes and in the *in vivo* gene delivered whole hearts abolishes the cardioprotection of BMPoC (Figure 6). Beclin 1 has been shown to play an essential role in autophagosome formation, while whether the I/R-induced impairment in autophagosome processing attributes to Beclin 1 abundance is not well known. As observed previously,^28, 30^ our results showed that knockdown of Beclin 1 resulted in restoration of autophagosome processing as indicated by reduction of LC3-II, autophagosome accumulation and P62 abundance, while beclin 1 overexpression abolished BMPoC-modulated autophogosome processing characterized by increases of LC3-II, GFP-LC3 puncta and P62 abundance (Supplementary Figures 1b and 5b). These results suggest that beclin 1 plays an important role in the regulation of autophagy during myocardial I/R. Beclin-1 has been shown to play a role in the cell death induced by I/R injury and their reduction through molecular strategies seems to protects the heart,^46^ while the similar results of an essential autophagy gene *Atg5* overexpression as those of Beclin 1 overexpression (Figure 6) to the cardioprotection of BMPoC (Supplementary Figure 6) suggest that this protection is mainly dependent on the autophagy regulation. Further investigations are needed to examine if there are any other kinds of effects responsible for cardioprotection of BMPoC. The increased level of Beclin 1 due to myocardial I/R at least contributes to the impairment of autophagic flux, whereas the down-regulation of Beclin 1 during I/R is required for the maintenance of normal autophagosome processing and against myocardial I/R injury. Therefore, we proposed that any agents suppressing the expression of Beclin 1 would have cardioprotection against I/R injury.

Myocardial I/R has been shown to induce an activation of PI3K/Akt signaling pathway, which could be further enhanced in the protected myocardium.^47, 48, 49^ This is confirmed in our study that the I/R-increased phosphorylation of Akt (ser473) is further enhanced by BMPoC. We further confirmed that the activated PI3K/Akt pathway contributes to the BMPoC-afforded cardioprotection by modulating autophagy. This is supported by following observations: (i) BMPoC activates Akt during I/R (Figure 7a); (ii) the improvement of BMPoC on I/R-altered autophagy dysfunction and Beclin 1 expression (Figure 7a), post-ischemic myocardial performance (Figure 7b) and cell death (Figure 2a) is abolished by a specific Akt inhibitor A6730; and (iii) A6730 abolishes the cardioprotective effects of Beclin 1-knockdown on I/R-induced infarction (Figure 8b). Thus, BMPoC confers cardioprotection by modulating beclin 1-dependent autophagy dysfunction through, at least partially, the activation of PI3K/Akt signaling pathway. Further studies are needed to investigate how the beclin 1 expression is regulated by PI3K/Akt signaling pathway.

Taken together, our data demonstrate that PoC with berbamine significantly improves LV functional recovery and limits infarct size. Such cardioprotection is at least partially mediated by the suppression of I/R-induced Beclin 1 expression and recovery of I/R-impaired autophagosome processing through the activation of PI3K/Akt signaling pathway. These findings not only reveal the potential therapeutic value of berbamine in the protection of myocardium from ischemia disease but also provide new insight to the understanding of molecular mechanisms of PoC.

## Materials and methods

### Animals

Adult male Sprague-Dawley (SD) rats from 250–300 g (Shanghai Slac Laboratory Animal Co. Ltd., Shanghai, China) were cared in accordance with the Guidelines for Care and Use of Laboratory Animals published by the US National Institutes of Health(NIH Publication, 8th Edition, 2011). Animal procedures were approved by the Institutional Review Board of the Institute of Health Sciences, Shanghai Institutes for Biological Sciences (Shanghai, China).

### I/R injury model in Langendorff-perfused rat hearts

Male SD rats were anaesthetized with sodium pentobarbital (45 mg/kg, i.p.). The hearts were rapidly excised and retrogradely perfused with Krebs–Henseleit buffer at 37 °C by using the Langendorff technique at a constant pressure of 80 mm Hg as previously described.^50^ LV pressure was monitored using a water-filled latex balloon connected to a pressure transducer (AD Instrument, Bella Vista, New South Wales, Australia) and inserted into the LV cavity achieving a LVEDP between 0 and 8 mm Hg. LVDP, LVEDP, maximal rates of pressure development over time (+dP/dtmax) and pressure decay over time (−dP/dtmax), and heart rate were monitored with PowerLab system (AD Instrument). Berbamine (Sigma, St. Louis, MO, USA), dissolved in deionized distilled water, was added to perfusate at final concentrations of 0, 10, 30, 100, 300 nM and a specific Akt1/2 inhibitor A6730 (2.5 *μ*M, Sigma) was added at the onset of reperfusion for 5 min. At the end of the reperfusion, the hearts were rapidly removed and frozen in liquid nitrogen for immunoblotting analysis.

### Infarct size estimation

The isolated rat hearts subjected to 30 min of ischemia followed by 2 h of reperfusion were frozen and the LV tissue was cut into 2-mm-thick slices. The slices were incubated in 1% w/v triphenyltetrazolium chloride (TTC, pH 7.4) for 15 min, and then fixed in 10% formaldehyde.^51^ Infarct size was calculated using Image-Pro-Plus software (Media Cybernetics), and the infarct area was expressed as a percentage of the LV area at risk.

### LDH activity

LDH activity in the coronary effluent was spectro-photometrically measured based on the oxidation of lactate at pre-ischemia and at 45 min of reperfusion as previously described.^49^

### Construction of recombinant adenoviruses

Recombinant adenoviruses expressing human *Beclin 1* (AdBeclin 1), short hairpin RNA of *Beclin 1* (AdshBeclin 1) and LacZ (AdLacZ) were prepared as described previously using the pAdEasy vector system (Qbiogene, Santa Ana, CA, USA).^31^ In brief, Beclin 1, and the LacZ sequence were cloned into pShuttle-CMV (Qbiogene), and the short heparin Beclin 1 (shBeclin 1) sequence (forward: 5′-AATTCGCAATTTGGCACGATCAATATGTGCTTTATTGATCGTGCCAAATT-GTTTTTTG-3′; reverse: 5′-GATCCAAAAAACAATTTGGCACGATCAATAAAGCACATATT-GATCGTGCCAAATTGCG-3′) was cloned into reconstituted pShutle-U6 (Qbiogene) and homologously recombined in bacteria BJ5183 with pAdeasy-1. The recombinant plasmids were propagated separately in HEK 293 cells. The titers of stocks measured by plaque assays were 2 × 10^10^ pfu/ml for AdLacZ, 1.2 × 10^10^ pfu/ml for AdBeclin 1 and 2 × 10^10^ pfu/ml for AdshBeclin 1. Adenovirus encoding human gene *Atg5* (2 × 10^10^ pfu/ml) was purchased from Vigene Bioscience Company, Jinan, China.

### *In vivo* adenoviral gene delivery

The surgical procedures and adenoviral delivery were carried out as previously described.^52^ In brief, the sodium pentobarbital (45 mg/kg, i.p.) anesthetized rats were performed thoracotomy. A 27 gauge needle containing 100 *μ*l of diluted adenovirus (3 × 10^10^ pfu/ml) or sterile saline was advanced from the apex of the LV to the aortic root. The aorta and main pulmonary arteries were clamped for 10 s distal to the site of the injector when the solution was injected, and then the chest was closed. The hearts underwent homodynamic studies at day 4 after the adenovirus injection.

### Isolation, culture and adenoviral infection of adult rat ventricular myocytes

Adult rat LV myocytes were isolated from adult rat hearts as previously described.^31, 53^ In brief, the freshly isolated heart was successively perfused with nominally Ca^2+^-free Tyrode's solution containing collagenase II (240 U/ml, Worthington Biochemical) and protease (0.12 mg/ml, Sigma) for 18–22 min. Finally, the cell suspension from left ventricles was rinsed with Tyrode's solution followed by a gradual increase in the Ca^2+^ concentration up to 1.25 mmol/l and 85% of isolated rod-shaped myocytes were Ca^2+^-tolerant. The isolated myocytes were then cultured with medium 199 (Sigma) supplemented with l-carnitine (2 mmol/l), N-2-mercaptopropionyl glycine (5 mmol /l), taurine (5 mmol/l), insulin (0.1 mmol/l), 2.5% FBS (Gibco, Carslbad, CA, USA), and penicillin/streptomycin (100 IU/ml). Adenoviral infection was performed as described previously.^31^ After 4 h of culture to achieve myocyte attachment, adenovirus-directed gene transfer was performed by adding a small volume of FBS-free medium 199 containing constructed adenovirus at a multiplicity of infection (the ratio of infectious virus particles to the number of cells being infected) of 100 for 2 h. All experiments were performed after 36 h of adenoviral infection.

### Simulated I/R in isolated cardiomyocytes and cell death measurement

A cellular model of simulated I/R (20 min/30 min) in ventricular myocytes was used as previously described.^54, 55^ In brief, myocytes were equilibrated in modified Krebs–Henseleit solution at 35 °C, pH 7.4. The solution was then switched to ischemic solution, containing (mmol/l): 123.0 NaCl, 8.0 KCl, 6.0 NaHCO_3_, 0.9 NaH_2_PO_4_, 0.5 MgSO_4_, 20.0 Na-lactate and 1.8 CaCl_2_, gassed with 95% N_2_ and 5% CO_2_ (pH 6.8) for 20 min followed by 30 min of reperfusion with modified Krebs–Henseleit solution. Microscopic methods were used to detect cardiomyocytes cell death based on distinct morphological changes, an approach used previously to quantify I/R-induced ARVM cell death.^56^

### Measurement of fluorescent LC3 puncta

Imaging studies for GFP-LC3 in cardiomyocytes were performed as previously described.^57^ In brief, cardiomyocytes cultured on coverslips were transduced with Ad-GFP-LC3. Two hours later, the culture media containing virus were replaced with fresh media. Simulated I/R experiments were performed at 24 h after transduction. After the various treatments, the cells were washed with PBS, fixed with 4% paraformaldehyde and viewed under Leica TCS SP2 confocal laser scanning microscope. The number of GFP dots was determined by manual counting of fluorescent puncta.

### Measurement of cell shortening

For measuring cell shortening, adult rat ventricular cardiomyocytes were simultaneously monitored through a fluorescence camera (Olympus, Tokyo, Japan) and myocyte contraction and Ca^2+^ recording system (IonOptix; Milton, MA, USA) as previously described.^31^

### Measurement of ΔΨm

ΔΨ_m_ were detected as described previously.^58, 59^ The cultured cardiomyocytes were incubated with TMRE (50 nmol/l, Molecular Probes) for 20 min. After loading, the cells were washed twice with Krebs–Henseleit solution. The cardiomyocytes attached to the coverslips were then transferred to a chamber mounted on the stage of a Leica TCS SP2 confocal laser scanning microscope and underwent simulated I/R perfusion. TMRE-loaded cells were excited at a maximum of 543 nm, and emitted light was collected from 552–620 nm. Twenty cells were randomly selected in each scan using a × 20 objective lens. Images were analyzed using LAS AF Lite software (Leica Microsystems, Atlanta, GA, USA).

### Protein preparation and Western blotting

Left ventricles were homogenized and the cells were lysed as described previously.^22^ In brief, ~100 mg of freeze-clamped LV tissues were homogenized at 4 °C with a homogenizer in 10 vols of lysis buffer containing 1% Triton X-100, 0.5% deoxycholate and 5 mmol/l 2-mercaptoethanol. Cell extracts were scraped into lysis buffer containing 20 mmol/l Tris-HCl (pH 7.4), 6 mM urea and 200 mmol/l potassium chloride with a protease inhibitor cocktail 3.6 mmol/l leupeptin, 2.1 mmol/l pepstatin A and 50 mmol/l phenylmethylsulfonylfluoride, followed by vigorous vortexing and cooling on ice for 15 min before 15 min of centrifugation at 12 000 × *g*. The concentration of proteins was determined using the Bradford method. The homogenates/lysates were stored at −80 °C. Tissue homogenates, cell lysates or immunoprecipitates were analyzed by standard immunoblotting analysis. Relative amounts of proteins were determined by specific antibodies against Beclin 1 (1 : 2000), anti-GAPDH (1 : 8000,), anti-phospho-Akt (Ser473, 1 : 2000), anti-Akt (1 : 2000,), anti-LC3 (1 : 2000) and anti-SQSTM1 (P62, 1 : 5000). Antibodies were purchased from Cell Signaling or Sigma. The immunoreaction was visualized with an enhanced ECL detection kit (Amersham Pharmacia Biotech, Buckinghamshire, England), then exposed to film and quantified with a video documentation system (Gel Doc 2000, Bio-Rad, Hercules, CA, USA).

### Real-time PCR

SYBR green (TOYOBO) real-time PCR (Q-PCR) for Beclin 1 was performed using cDNA generated from total RNA extracted from LV tissue. Beclin 1 primers are as follows: sense, 5′-ATCCTGGACCGAGTGACCATTC-3′ and antisense, 5′- TCTCCTGAGTTAGCCTCTTCCTCC -3′.

### Data and analysis

Data were expressed as means±S.E.M. For the statistical analysis, a one-way ANOVA, when significant, followed by a Dunnett *post hoc* test was applied to *ex vivo* experiment of cardioprotective effects of BMPoC, comparison of infarct size between the control I/R and BMPoC-treated I/R groups and examination of Beclin 1 expression during I/R. Other data were analyzed with two-way ANOVA or repeated ANOVA, when significant, followed by a Bonferroni *post hoc* test. All analyses were performed using GraphPad Prism version 5.0 software (CA, USA). A *P*-value<0.05 was considered statistically significant.
