# Supplementary material for: Berbamine postconditioning protects the heart from ischemia/reperfusion injury through modulation of autophagy
Source: Cell Death Dis. 2017 Feb 2;8(2):e2577–. doi: 10.1038/cddis.2017.7 (PMC5386498; doi:10.1038/cddis.2017.7)

**Supplementary data**

**Berbamine postconditioning protects the heart from ischaemia/reperfusion injury through modulation of autophagy**

Yanjun Zheng^1,2^, Shanshan Gu^1^, Xuxia Li^1^, Jiliang Tan^1^, Shenyan Liu^1^, Yukun Jiang^1^, Caimei Zhang^1^, Ling Gao^1^ and Huang-Tian Yang^1,2^*

^1^Key Laboratory of Stem Cell Biology and Laboratory of Molecular Cardiology, Institute of Health Sciences, Shanghai Jiao Tong University School of Medicine (SJTUSM) & Shanghai Institutes for Biological Sciences (SIBS), Chinese Academy of Sciences (CAS), Shanghai, China

^2^Heart Center, Shanghai Jiaotong University Affiliated Sixth People's Hospital, Shanghai, China

**Running title: Cardioprotection of berbamine postconditioning**

***Correspondence author:**

Huang-Tian Yang, Ph.D., Institute of Health Sciences, 320 Yue Yang Road, Biological Research Building A, IHS Mail Box 115, Shanghai, 200031, China. Phone/Fax: +86-21-54923280; e-mail: htyang@sibs.ac.cn

**Supplementary figure legends**

**Supplementary Figure S1.** Immunoblots and analysis data of LC3 and P62. **a.** Representative immunoblots (upper panel) and averaged immunoblot data (lower panel) of LC3 and P62 in adult cardiomyocytes with or without simulated I/R and 20μM chloroquine (CQ); **b.** Representative immunoblots (upper panel) and averaged immunoblot data (lower panel) of LC3 and P62 in cardiomyocytes adenovirally infected with AdBeclin 1 and Adsh Beclin-1 with or without BMPoC. n = 5; data were presented as means ± SEM. ^*^*P* < 0.05 between the indicated groups. ns indicates not significant.

**Supplementary Figure S2.** Beclin 1 expression during I/R with or without BMPoC. **a**, Q-PCR analysis of Beclin 1 mRNA during simulated I/R (sI/R) with or without BMPoC in cardiomyocytes; **b**, Representative immunoblots (left panel) and quantitative analysis (right panel) of Beclin 1 expression during I/R with or without BMPoC in rat left ventricular; **c**, Q-PCR analysis of Beclin 1 mRNA during I/R with or without BMPoC in rat left ventricular. n = 5 each; data were presented as means ± SEM. R45, 45 min of reperfusion. ^*^*P* < 0.05 vs. pre-ischaemia control; ^#^*P <* 0.05 vs. the control I/R (R45) group.

**Supplementary Figure S3.** Effects of AdBeclin 1- or AdshBeclin 1-infection on the Beclin 1 expression in the cardiomyocytes and hearts. **a**, Representative immunoblots (upper panel) and quantitative analysis (lower panel) of Beclin 1 protein expression in the cardiomyocytes after 36 h of infection; **b**, Representative immunoblots (upper panel) and quantitative analysis (lower panel) of Beclin 1 protein expression in the heart after 3 days of infection. GAPDH was used as an internal reference. n = 5 each; data were presented as means ± SEM. **P* < 0.05 vs. AdLacZ controls.

**Supplementary Figure S4.** Representative immunofluorescence images (upper panel) and analysis (lower panel) of cardiomyocytes expressing green fluorescent protein (GFP)–light chain-3 (LC3) and LC3 puncta (indicator of autophagosomes) in cardiomyocytes in normal condition (pre-ischaemia). Scale bar = 10 μm. The statistical analysis was conducted on the averaged value for each rat heart and n = 5; data were presented as means ± SEM.

**Supplementary Figure S5.** Effects of Beclin 1 knockdown and overexpression on the cardiac functional index of pre-ischaemic phase and heart rate in isolated rat hearts subjected to 30 min no-flow global ischemia followed by 45 min of reperfusion with or without BMPoC. **a-d**, LVDP, LVEDP, and ±dP/dt max during pre-ischemic phase; **e**, Analysis of heart rate at pre-ischemia and 45 min of reperfusion (R45). n = 5 hearts each; data were presented as means ± SEM.

**Figure S6.** Effects of Atg5 on LV performance and cell death in isolated rat hearts subjected to I/R with or without BMPoC. **a-d,** Effects of Atg5 overexpression on the post-ischaemic recovery of LVDP (**a**), LVEDP (**b**), +dp/dt max (**c**) and -dp/dt max (**d**) with or without BMPoC; **e**, Effects of Atg5 overexpression on the LDH activity in the coronary effluent with or without BMPoC; **f**, Representative images (upper panel) and analysis (lower panel) of infarct size in perfused I/R (30 min/2 h) hearts. R45, 45 min of reperfusion. n = 4-6 hearts each; data were presented as means ± SEM. ^*^*P <* 0.05 vs. the pre-ischaemic AdLacZ group; ^#^*P <* 0.05 vs. the AdLacZ-infected I/R hearts; ^†^*P <* 0.05 vs. the AdLacZ-infected hearts with BMPoC.

**Figure S7.** Heart rate at 5 min prior to ischemia (Pre-ischemia) and 45 min of reperfusion (R45) with and without BMPoC in the presence and absence of a specific Akt inhibitor A6730 (2.5 μM). Data were presented as means ± SEM.


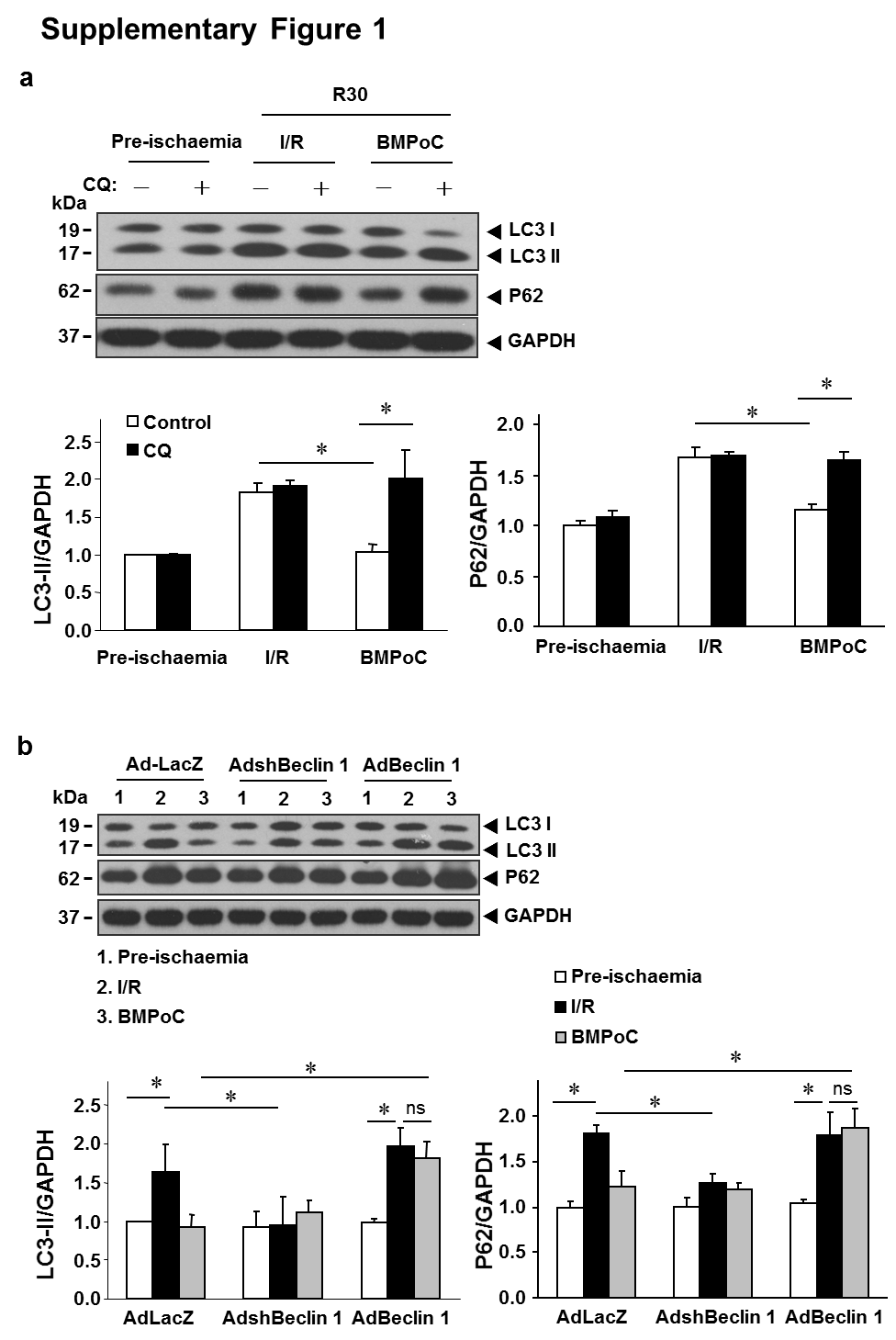


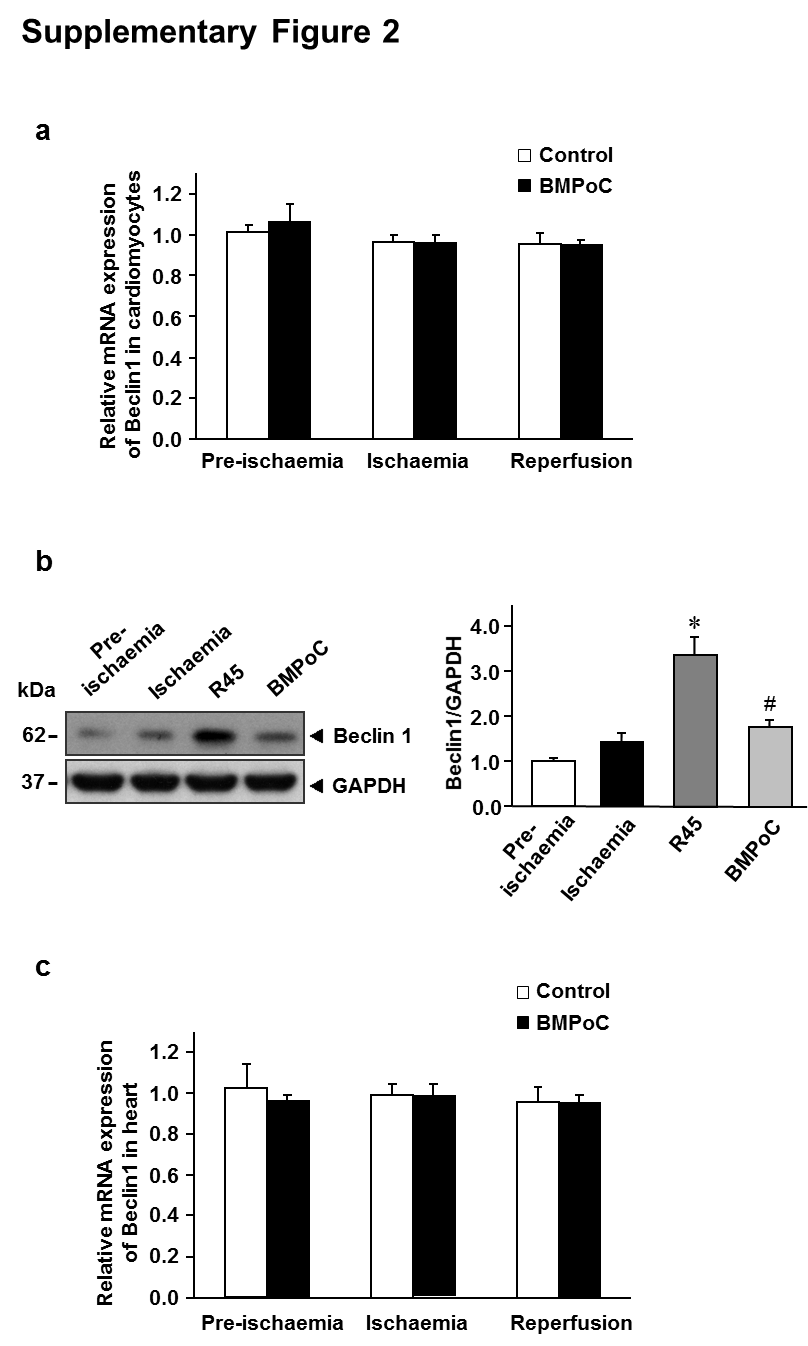


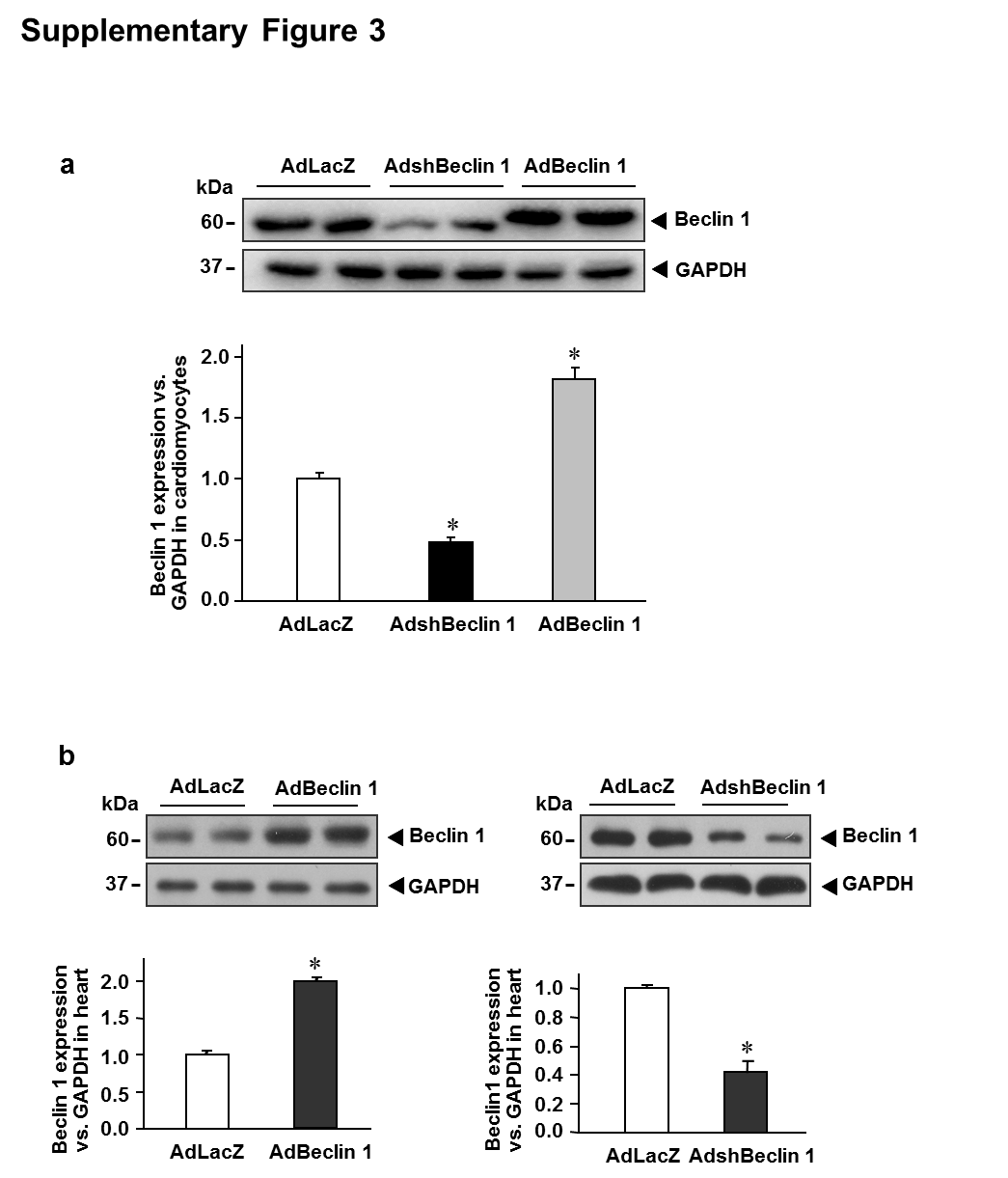


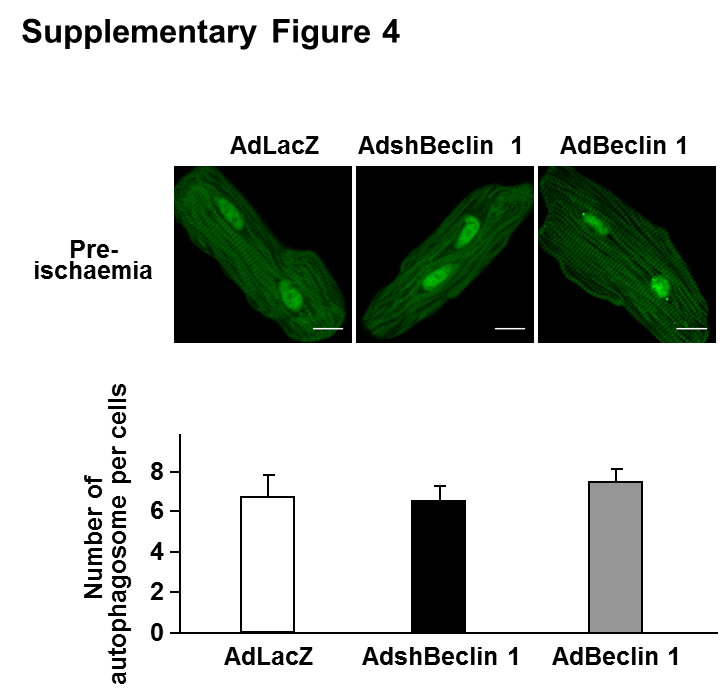


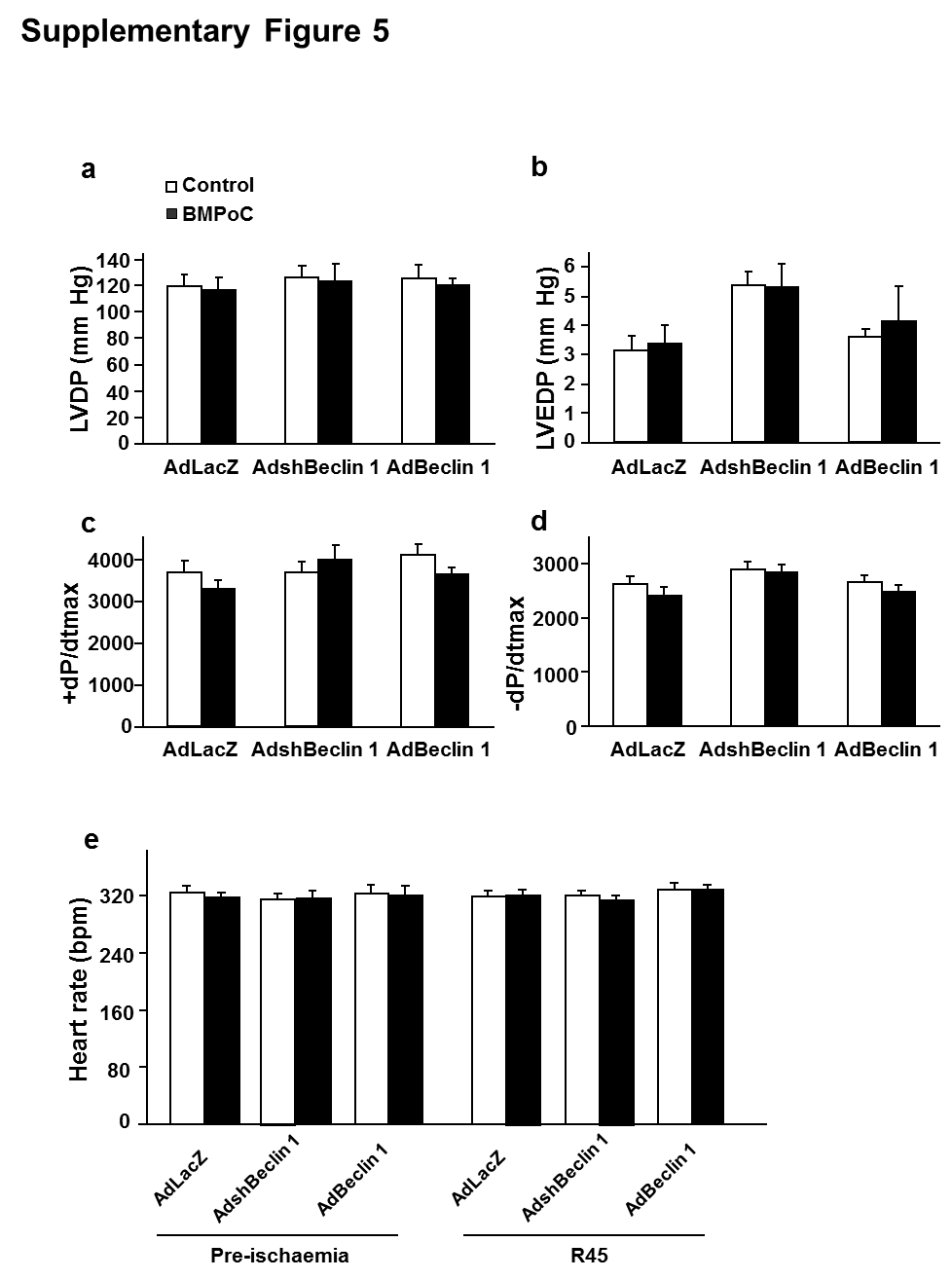


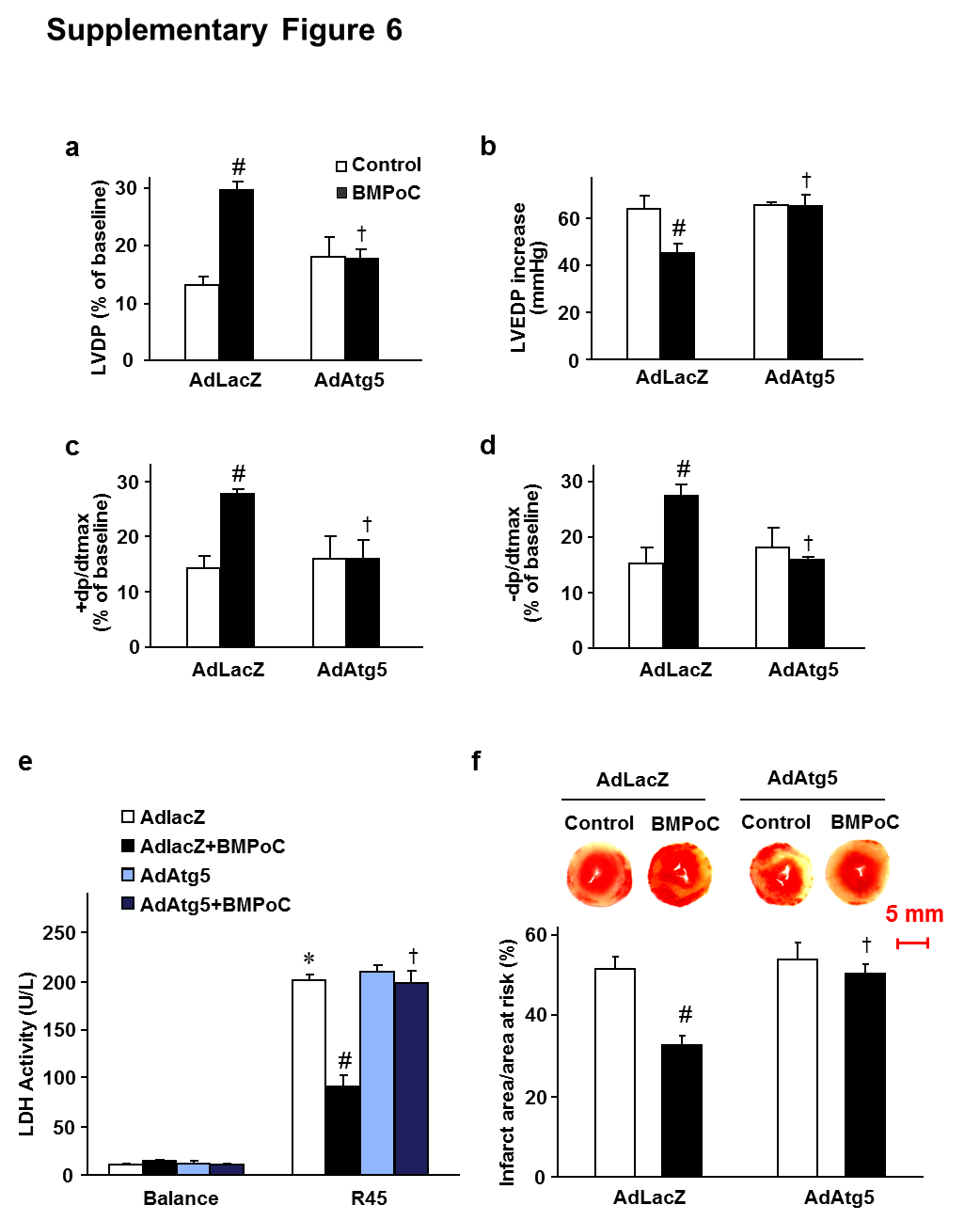


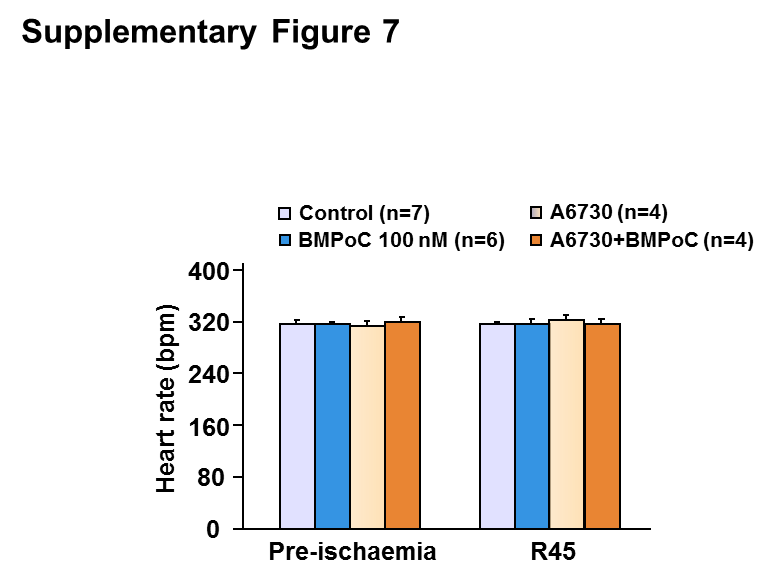

Supplement: Supplementary Information [file cddis20177x1.doc]
